# Working in disadvantaged communities: What additional competencies do we need?

**DOI:** 10.1186/1743-8462-6-10

**Published:** 2009-04-24

**Authors:** Elizabeth Harris, Mark F Harris, Lynne Madden, Marilyn Wise, Peter Sainsbury, John MacDonald, Betty Gill

**Affiliations:** 1 Centre for Health Equity Training, Research and Evaluation, University of New South Wales, Sydney, NSW, Australia; 2 Centre for Primary Health Care and Equity, University of New South Wales, Sydney, NSW, Australia; 3 Current address: Australasian Faculty of Public Health Medicine, Royal Australasian College of Physicians, Sydney, NSW, Australia; 4 Division of Population Health, NSW Department of Health, NSW, Australia; 5 Department of Public Health, University of Sydney, Sydney, NSW, Australia; 6 Division of Population Health, Sydney South West Area Health Service, Sydney, NSW, Australia; 7 Social Justice Social Change Research Centre, University of Western Sydney, Sydney, Australia; 8 College of Health and Sciences, University of Western Sydney, Sydney, Australia

## Abstract

**Background:**

Residents of socioeconomically disadvantaged locations are more likely to have poor health than residents of socioeconomically advantaged locations and this has been comprehensively mapped in Australian cities. These inequalities present a challenge for the public health workers based in or responsible for improving the health of people living in disadvantaged localities. The purpose of this study was to develop a generic workforce needs assessment tool and to use it to identify the competencies needed by the public health workforce to work effectively in disadvantaged communities.

**Methods:**

A two-step mixed method process was used to identify the workforce needs. In step 1 a generic workforce needs assessment tool was developed and applied in three NSW Area Health Services using focus groups, key stakeholder interviews and a staff survey. In step 2 the findings of this needs assessment process were mapped against the existing National Health Training Package (HLT07) competencies, gaps were identified, additional competencies described and modules of training developed to fill identified gaps.

**Results:**

There was a high level of agreement among the AHS staff on the nature of the problems to be addressed but less confidence indentifying the work to be done. Processes for needs assessments, community consultations and adapting mainstream programs to local needs were frequently mentioned as points of intervention. Recruiting and retaining experienced staff to work in these communities and ensuring their safety were major concerns. Workforce skill development needs were seen in two ways: higher order planning/epidemiological skills and more effective working relationships with communities and other sectors. Organisational barriers to effective practice were high levels of annual compulsory training, balancing state and national priorities with local needs and giving equal attention to the population groups that are easy to reach and to those that are difficult to engage. A number of additional competency areas were identified and three training modules developed.

**Conclusion:**

The generic workforce needs assessment tool was easy to use and interpret. It appears that the public health workforce involved in this study has a high level of understanding of the relationship between the social determinants and health. However there is a skill gap in identifying and undertaking effective intervention.

## Introduction

Residents of socioeconomically disadvantaged locations are more likely to suffer poorer health than residents of socioeconomically advantaged locations [1]; this has been comprehensively mapped in Australian cities [2]. The causes of this concentration of disadvantage are complex, with roots in the historical development of a location, the availability of low cost housing, changes to the economic base of an area that leads to spiralling disadvantage over time and lack of essential economic and social infrastructure such as employment, transport and services [3].

While debate continues about the interaction between people (composition) and context (the nature of the location) in creating poorer health outcomes, there is little dispute about the significant spatial patterning of health inequality [4]. These inequalities present a challenge for the public health workers based in or responsible for improving the health of people living in disadvantaged localities.

In 2001, with funding through the Public Health Education and Research Innovation Grants Program, we established a consortium of organisations to develop a practical approach to identifying the workforce development needs of a local health workforce to enable them to work more effectively in disadvantaged communities. In undertaking this work we chose to look at specific geographical locations that were reported as experiencing high levels of social and economic disadvantage. However, in doing so we were cognisant of the fact that there are many ways in which disadvantaged locations can be defined; that in many of these locations there are people who may experience other forms of disadvantage related to disability or ethnicity; that many people with socioeconomic disadvantage may live in pockets outside such readily recognised disadvantaged locations; and that not all people living in readily identified disadvantaged locations are socially and economically disadvantaged.

The challenge as we saw it was to ensure that the public health workforce in disadvantaged communities is competent to address the needs of these communities. We assessed that there is little formal preparation of the public health workforce to work in these locations. We sought ways therefore, in which the competencies needed for this work could be obtained through recognised training in the vocational education sector undertaken within the National Health Training Package framework or in the higher education sector. Using appropriate existing training pathways was considered to be more sustainable than developing a completely separate process for identifying, developing and delivering the skills and knowledge required.

This chapter describes the processes through which the necessary workforce competencies were identified; how these were mapped against existing competencies for working in population health within the National Health Training Package; and provides an outline for a proposed learning module that will be piloted in the higher education sector late in 2009.

For the purposes of this study a broad definition of the public health workforce was taken and included staff from the health sector working in: Aboriginal health; alcohol and other drugs; child protection; community health; community nursing; community nutrition; community development; dental and oral health; divisions of general practice; environmental health; health planning; health promotion; learning and development management; mental health; multicultural health; primary health care; public health and women's health. It did not include workers from other sectors who contribute to public health but who are not part of the health workforce, such as local government and housing workforces. Locational disadvantage in health can be understood as the extent to which health is influenced by the interrelationship between the area in which people live (the context) and the people who live there (composition of the area).

## Methods

Two literature reviews were conducted. The first, Workforce development needs assessment: an annotated bibliography [Bartlett M, Madden L, Wise M. Workforce development needs assessment: an annotated bibliography. Centre for Health Equity Research Training and Evaluation, UNSW, Unpublished] sought to identify generic workforce needs assessment tools and processes that could be applied to the public health workforce. This literature review informed the generic needs assessment process that was developed.

The second literature review, Location, disadvantage and health: a review of the literature, sought to identify evidence of the relationship between location, disadvantage and health and to identify effective community-based interventions that had been undertaken in disadvantaged communities [5]. This was done to assist in identifying competencies, based on what was known of effective interventions in disadvantaged communities as the basis of what the public health workforce need to know and be able to do.

A two-step mixed method process was used to identify the workforce needs. In Step 1 a workforce needs assessment was undertaken in three NSW area health services. In Step 2 the findings of this needs assessment process were mapped against the existing

National Health Training Package (HLT07) competencies, gaps were identified, additional competencies described and modules of training developed to fill identified gaps.

### Step 1: Workforce needs assessment

At the core of undertaking a workforce needs assessment is an understanding of the nature of the work to be undertaken and the organisational context within which the work will occur [5,6]. This means the knowledge, skills and attributes [7] required by a workforce are defined in terms of the work to be performed and the outcomes to be achieved rather than by a 'professional role'. Based on this understanding, the project sought to answer the following questions:

• What is the issue to be addressed?

• What is the work that needs to be done?

• What is the best way to address these issues?

• What is the capacity of the organisation and workforce to do this work?

• What are the best ways to address these issues at the workforce and organisational level?

This generic workforce needs assessment framework was applied in three area health services (AHS): a large outer metropolitan AHS; an urban/rural AHS; and a rural AHS. Organisational responses to the questions in the framework were sought from service managers, and workforce responses were sought from public health teams and individual workers in recognition that all the required capacity may not need to exist within single workers but could be spread across and within teams.

#### Data Collection

##### Survey instruments

Qualitative data was collected from managers and teams through a series of focus groups and semi-structured interviews based on the generic needs assessment questions. Twenty-five managers were interviewed across the three AHSs. In all AHSs the managers of health promotion, public health, community health, multicultural health, Aboriginal health, and human resources were interviewed, as well as other senior staff.

Focus groups were held with six teams in two AHSs. Participants represented community nutritionists, health promotion and community health workers. An additional six interviews were held by telephone with individual workers who were unable to attend the group meeting.

Quantitative data was collected from staff using a survey based on a series of standardised questions that had been used previously in other public health workforce needs assessment processes [8-10]. Participation was voluntary and responses were anonymous.

The surveys were sent to 200 workers who were nominated by their public health managers as working in disadvantaged communities; 84 (42%) responded. There were almost equal numbers of respondents from public health units, health promotion units and community health.

##### Data analysis

The semi-structured interviews and focus groups were taped and transcribed. Themes and issues contained in the data were identified and coded using NVivo version 2.0. Data from the surveys were entered into Excel and then transferred to SPSS for Windows version 11 for descriptive analysis. Open-ended responses were coded into categories. Categories were reviewed by another staff member to check reliability.

Data from both the qualitative and quantitative methods were integrated to answer the key workforce needs assessment questions.

### Step 2: Identification of workforce competencies

Following the workforce needs assessment we commissioned the consultants who were responsible for developing the population health component of the National Health Training Package to compile a complete list of the required skills, knowledge and attitudes for working in disadvantaged communities. The consultants were commissioned to undertake specific tasks on a contractual basis and have no formal links with the investigators.

The list was compiled by analysing the findings of the second literature review [5] that examined the evidence for effective interventions in disadvantaged communities and the report of the analysis of the workforce needs assessment [11]. Competencies that the consultants identified were then mapped against the skills, knowledge and attitudes specified as pre-requisites to successful job performance within the Population Health qualifications of the National Health Training Package (HTL07) [12]. The sources from which a specific competency was identified (i.e. literature review, focus groups, interviews and survey) were noted.

Following this, the consultants were commissioned to develop the module descriptors for a short course designed to develop the identified competencies not currently accommodated within the existing (HTL07) training package framework [13].

## Findings

### Step 1: Workforce needs assessment

#### What is the problem to be addressed?

There was a high level of agreement between service managers and teams on the range of problems to be addressed in disadvantaged populations. All three AHSs were experiencing changes in demographic structures: some people moving into the area then needed to travel long distances to work; an increase in retirees; and low income residents being pushed to the edges of settlement with resultant social isolation. Lack of infrastructure including transport, employment, human services (including health services) and poor amenity in the communities were consistently mentioned as problems. Managers generally saw these communities as having the same health problems as the community as a whole but were supported by less service infrastructure. In particular, they were concerned at the lack of services to enable mandatory child protection and domestic violence reporting.

Public health units reported that while their work priorities were largely dictated by national and state imperatives, they were trying to influence the planning of new communities – with some success.

Managers reported a tension between providing child and family services and aged services for groups who were easy to reach and comparatively well-off and providing the same services for disadvantaged populations. More than one team expressed concern that identification of disadvantaged communities would lead to negative images of these communities that were often reinforced in the media.

The survey respondents identified four main problem areas: problems created by the social determinants of health (such as unemployment, crime, lack of transport); the poor health status of people in these communities; service delivery issues; and spatial and environmental issues such as poor amenity and rubbish.

#### What is the work that needs to be done?

Both managers and teams had difficulty in untangling the difference between the problems to be addressed and the work that needed to be done. Participants in the focus groups identified improving school retention rates, improving local amenity, community engagement and partnerships with other agencies as potential areas of work.

Survey respondents identified a different set of actions that included implementing work already identified in existing plans and priorities, use of local health data and community needs assessments, and consultation with other stakeholders (including residents). They also stated that much of their work was reactive to changes in local conditions.

All three groups of respondents primarily focused on the process for identifying the work to be done rather than details of the work itself.

#### What is the best way to address the issues?

Managers and teams identified comprehensive needs assessments as a key basis for intervention. These needs assessments generally were seen as involving consultation with the local community, other government departments, non-government organisations and community groups. Building trust and rapport with the local community was considered critical to successful intervention by the managers, teams and survey respondents. Managers and survey respondents also mentioned tailoring or adapting standard approaches to make them acceptable and appropriate as being important in undertaking interventions.

A range of barriers to effective implementation of interventions was also identified by managers and teams; these included the demand of health sector funding bodies for interventions that are innovative or pilots, with heavy reliance on short-term funding and limited capacity (even when the intervention was effective) to be integrated into mainstream services. Lack of time to develop and implement programs was also seen as problematic, as were different perceptions of success by different stakeholders.

A major tension mentioned by teams was balancing differences between community priorities and priorities of the health services (which were often set at state and national levels). A similar problem was identified by Ridoutt et al. in a study of the public services of a large outer metropolitan area health service [6].

#### What is the capacity of the organisation and workforce to do this work?

Managers were concerned at the difficulties in recruiting and retaining staff to work in disadvantaged communities. They spoke of new graduates working in the AHS until they had enough experience to be employed in more competitive job markets. They also noted that where the workforce had become entrenched there was resistance to new ways of working, especially in taking more proactive approaches such as home visiting or outreach services. Managers saw skill development in two ways: some spoke of the need for higher order planning, research and epidemiological skills, while others saw skill development more in terms of higher order communication skills with flexibility in responding to community needs. Debriefing of inexperienced workers and safety were seen as being areas that require attention.

The majority of teams felt that the levels of skill and experience needed to work in disadvantaged areas were higher than for other communities; new graduates expressed concern that they did not have the required skill level. Experience in working in these communities was seen as important in preventing "burn out" in staff.

Experienced staff were able to manage the stress of working with a variety of inter-sectoral partners, dealing with the service structures and other government procedures, meeting sometimes conflicting community demands and dealing with the longer timeframes needed when working with disadvantaged communities and clients.

The survey assessed the capacity of the workforce at the team level in recognition that all the skills that may be required to work effectively in disadvantaged communities may not be found in one person. Survey respondents were asked to rate their team's ability against the core functions of public health. A large proportion of respondents rated their team's ability to perform assessments or address environmental issues as 'very good' or 'excellent'. In contrast, the ability of respondents' own teams to address the needs of vulnerable groups and communities was rated as 'somewhat weak' or 'poor' by a half and a third of the respondents respectively.

Almost two-thirds of the sample believed that personal security was an issue of concern for public health workers in disadvantaged communities. Half of the survey respondents, who came from a wide range of disciplinary backgrounds, reported that they spent more than 60% of their time working on public health issues. Three-quarters of respondents reported that they spent up to 60% of their time working on health issues in specific neighbourhoods or communities or working with clients from disadvantaged backgrounds.

#### What workforce and organisational development is needed to do this work?

Managers – especially in rural areas – expressed concern that the high levels of annual mandatory training left them with limited budgets to provide additional training opportunities. They identified the lack of suitable training, and its cost, as preventing uptake of training in new and innovative areas. These issues were supported by teams who also mentioned the importance of mentors.

The majority of survey respondents reported that they were interested in attending public health, statistics, research, and evaluation courses over the next 1 to 3 years. Others were interested in management and leadership and train-the-trainer courses.

Key organisational development needs identified included the recruitment and retention of staff and the provision of support. Some managers felt it was time to see locational disadvantage work as "core business" and not as a series of pilot projects.

Major tables from the report are included as Additional file 1.

### Step 2: Identification of workforce competencies

The skills, knowledge and attitudes needed to work effectively in disadvantaged communities were found to be both generic (e.g. the ability to work collaboratively with others is required in many domains of work) and specific (e.g. understanding the difference between contextual and compositional factors within disadvantaged communities). Most of the skills, knowledge and attitudes cited by at least two data sources were generic in nature. However, the skills, knowledge and attitudes that were specific to disadvantaged communities were often identified by only one data source and often pointed to the need to include new ways of thinking about or working in disadvantaged communities (such as monitoring the underlying determinants of health).

The identified competencies were then mapped to the competencies in the National Health Training Package (HLT07). Many of the 30 generic competencies we had identified matched 'perfectly' or 'sufficiently' to units of competency already detailed in the current Training Package; for example, 'conducting needs assessments' and 'building relationships and partnerships'. However, 10 of the 12 competencies that we identified as specific and critical to working in disadvantaged communities matched 'poorly' or 'not at all' (that is there was no suitable unit of competence) [12]. The report of the mapping is included as Additional file 2.

The research that informed the mapping exercise was then used to develop module descriptors for a course on working in disadvantaged communities within the vocational sector. This course is planned to have three modules: identification of disadvantaged communities; assessment of those locational factors (contextual and compositional) that impact on health; and development and evaluation of interventions to improve health in disadvantaged communities (see Appendix 1).

## Discussion

The overall aim of this work was to identify the competencies required by individual public health workers, teams and organisations to work effectively in and with disadvantaged communities. From the beginning our intent was for the competencies we identified to be driven by the nature and scope of the work that needs to be done rather than by the needs of the professional workers and/or the organisations in which they worked. To accomplish these aims we developed a generic workforce needs assessment framework, consisting of five broad questions, which we used in interviews, focus groups and a survey in three area health services to identify the competencies public health workers and managers felt were essential for working in disadvantaged communities; second, we conducted a literature review to identify the competencies that previous authors had identified as necessary for working in disadvantaged communities; third, we compiled a single list of competencies from the literature and our own research that we compared with the competencies in the National Health Training Package; and fourth, we developed three training modules for a short course to provide training for public health workers in the competencies not already covered by the Package.

**Figure 1:**
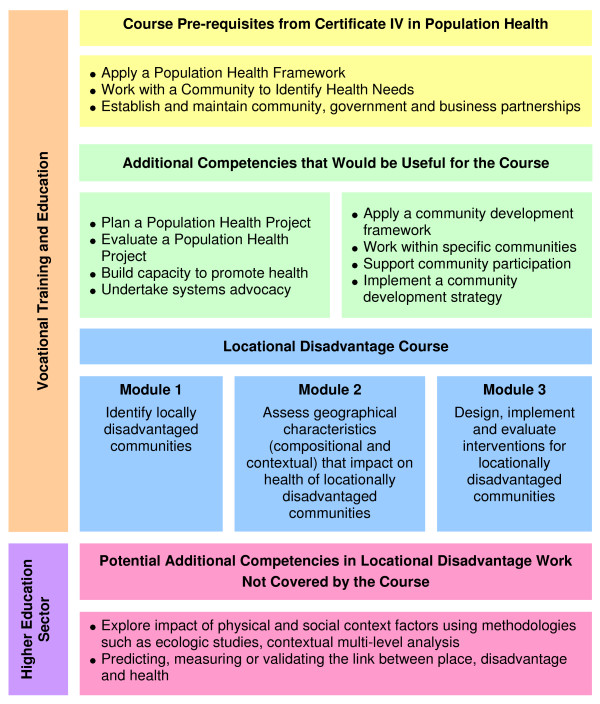
**Where the Locational Disadvantage Course Fits with the Certificate IV in Population Health**.

The successes of this project are that:

1. We engaged a wide range of public health staff and managers in metropolitan, semi-rural and rural areas in the identification of competencies required to work with disadvantaged communities to promote, maintain and protect their health.

2. The generic workforce needs assessment framework performed well and has been successfully used in other workforce needs assessment processes.

3. We identified relevant competencies for working with disadvantaged communities.

4. We have developed a short course to provide training in those competencies not already covered in an existing training package (see Figure 1 and Appendix 1).

However, it is important to recognise that only a small proportion of the public health workforce was involved in the study and many groups were under-represented; for example, general practitioners. Also, we do not know how representative of the public health workforce the survey respondents were as these people were identified by their managers. And crucially, we have not yet delivered the course and so have not been able to evaluate whether public health workers perceive a need for it, and if so, how successfully we can meet that need, let alone whether the training has any effect in improving outcomes in disadvantaged communities.

Within these limits we have identified some important issues. First, there was a high level of awareness within the public health workforce of the social determinants of health but there was much less awareness of and confidence in describing what can be done by local health services. This difficulty appeared to be at both a theoretical and a practical level.

Second, recognition by health professionals of the poorer health outcomes for people living in disadvantaged communities was at a high level, and evidence existed of a high level of investment by health services in working with people from disadvantaged communities, at the individual, family and community levels. However, much of this work is based on the adaption of existing mainstream health programs and not on evidence of what is likely to work in these communities.

Third, both managers and teams identified overcoming organisational and resource constraints and difficulties recruiting and retaining the workforce in disadvantaged areas as their most important organisational priorities. The need for a clear mandate from senior staff to invest resources in disadvantaged communities and the importance of moving existing programs from pilot, short-term to mainstream interventions was also stressed. However, this study did not extensively explore the organisational barriers and facilitators to working in disadvantaged communities and we hope this will be the focus of a further study.

This study demonstrates the development of public health training based on the results of systematic assessment of the work to be performed, the outcomes desired and the organisational context of the work rather than on the needs of professional groups or organisations.

## Competing interests

The authors declare that they have no competing interests.

## Authors' contributions

EH had the original idea for the project and took direct responsibility for the project's implementation. All authors participated in the development of the protocol, oversight of the project, and drafting and reviewing the chapter. All authors take responsibility for the chapter.

## Appendix 1: Training modules

### Module 1: Identify disadvantaged communities

The module is designed to provide an overview of the concepts that are particularly relevant to working in disadvantaged communities, identifying the dimensions and meanings of locational disadvantage and the existing means to confirm its presence and measure its likely impact on health. The module's purpose is to provide a theoretical grounding in locational disadvantage and to develop skills important to determining and advocating a locational disadvantage approach.

### Module 2: Assess community characteristics that impact on the health and wellbeing of disadvantaged communities

The module is designed to focus on ways in which the impact on health of features of a geographical community (both contextual and compositional) can be systematically identified and analysed. The module's purpose is to provide the conceptual tools and skills to properly understand aspects of working within a specific community that need to be addressed.

### Module 3: Design, implement and evaluate interventions

The purpose of this module is to assist learners to apply public health competencies in the design, implementation and evaluation of health interventions specific to disadvantaged communities through the use of context-specific tools and processes.

## Supplementary Material

Additional file 1 **Selected tables**. Selected Tables from *Locational Disadvantage: Focusing on Place to Improve Health *[9]. Click here for file

Additional file 2 **Competency identification**. The report on mapping of competency identification. Click here for file
