# Supplementary material for: Working in disadvantaged communities: What additional competencies do we need?
Source: Aust New Zealand Health Policy. 2009 Apr 24;6:10. doi: 10.1186/1743-8462-6-10 (PMC2684114; doi:10.1186/1743-8462-6-10)
Supplement: Additional file 1 — Selected tables. Selected Tables from Locational Disadvantage: Focusing on Place to Improve Health [9]. [file 1743-8462-6-10-S1.doc]

Additional file 1: Selected Tables from *Locational Disadvantage: Focusing on Place to Improve Health* [11]

Table 1: Respondent Characteristics

| Characteristic | N | Proportion (%) | Significant Differences by Workforce Segment[[1]](#footnote-2) |
| --- | --- | --- | --- |
| *Gender* |  |  | No |
| Male | 26 | 31.0% |  |
| Female | 58 | 69.0% |  |
| *Age* |  |  | No |
| <2 | 4 | 4.9% |  |
| 26-35 | 19 | 23.2% |  |
| 36-45 | 34 | 41.5% |  |
| 46-55 | 19 | 23.2% |  |
| 56+ | 6 | 7.3% |  |
| *Work Environment* |  |  | No |
| Area Health Service | 49 | 60.5% |  |
| Hospital | 3 | 3.7% |  |
| Community Health Centre | 13 | 16.0% |  |
| Specialist Unit or Team | 16 | 19.8% |  |
| *Position* |  |  | No |
| Management | 20 | 24.4% |  |
| Staff Member | 62 | 75.6% |  |
| *Length in Current Position* |  |  | No |
| <2 years | 40 | 47.1% |  |
| 2-4 years | 23 | 27.1% |  |
| 5-9 years | 10 | 11.8% |  |
| 10+ years | 12 | 14.1% |  |
| *Highest Qualification Completed* | | | No |
| Secondary/TAFE | 8 | 9.3% |  |
| Bachelor Degree | 26 | 30.2% |  |
| Postgraduate Certificate/  Diploma | 28 | 32.6% |  |
| Masters Degree/PhD | 24 | 27.9% |  |
| *Workforce Segment* |  |  | No |
| Public Health | 29 | 33.7% |  |
| Community Health | 29 | 33.7% |  |
| Health Promotion | 28 | 32.6% |  |

Table 2: Main Issue Categories Cited by Workers

| Issue Categories | **Number of Times Referred To[[2]](#footnote-3)** | **Number of Respondents Referring To** | **Proportion of Respondents Referring To** |
| --- | --- | --- | --- |
| Social determinants of health[[3]](#footnote-4) | 106 | 52 | 60.5% |
| Service Delivery Issues[[4]](#footnote-5) | 77 | 45 | 52.3% |
| Poor Health of the Communities[[5]](#footnote-6) | 49 | 26 | 30.2% |
| Spatial and Environmental Issues[[6]](#footnote-7) | 30 | 23 | 26.7% |
| Other | 9 | 9 | 10.5% |
|  |  |  |  |
| Total | 271 | 86 | 100.0% |

Table 3: Method of Identifying Work Needed

| Issue Categories | **Number of Times Referred To[[7]](#footnote-8)** | **Number of Respondents Referring To** | **Proportion of Respondents Referring To** |
| --- | --- | --- | --- |
| Existing plans, priorities and directives | 71 | 46 | 53.5% |
| Data and needs analysis | 58 | 43 | 50.0% |
| Consultations | 51 | 35 | 40.7% |
| Reactive prioritising | 13 | 10 | 11.6% |
|  |  |  |  |
| Total | 193 | 86 | 100.0% |

Table 4: Interventions Used in Communities

| Issue Categories | **Number of Times Referred To[[8]](#footnote-9)** | **Number of Respondents Referring To** | **Proportion of Respondents Referring To** |
| --- | --- | --- | --- |
| Community & Relationship Building | 96 | 52 | 60.5% |
| Standard Approaches | 38 | 25 | 29.1% |
| Other | 19 | 16 | 18.6% |
| Change Based | 8 | 7 | 8.1% |
|  |  |  |  |
| Total | 161 | 86 | 100.0% |

Table 5: Public Health Partners

| Issue Categories | **Number of Times Referred To[[9]](#footnote-10)** | **Number of Respondents Referring To** | **Proportion of Respondents Referring To** |
| --- | --- | --- | --- |
| Government | 91 | 46 | 53.5% |
| Communities | 28 | 18 | 20.9% |
| Other organizations (incl. Divisions of General Practice) | 19 | 14 | 16.3% |
| NGOs | 17 | 13 | 15.1% |
| No partners | 3 | 3 | 3.5% |
|  |  |  |  |
| Total | 158 | 86 | 100.0% |

Table 6: Organisational Environment and Support

| **Statement** | **Total Number of Respondents (N)** | **Proportion of Respondents Agreeing[[10]](#footnote-11)** | **Significant Differences by Workforce Segments[[11]](#footnote-12)** |
| --- | --- | --- | --- |
| I have the necessary experience to respond to the issues faced by clients from disadvantaged communities | 82 | 78.0% | Yes, community health agreed more[[12]](#footnote-13) |
| I have the necessary knowledge to help clients from disadvantaged communities | 82 | 78.0% | No |
| I do not have many of the skills necessary to address issues faced by clients from disadvantaged areas | 81 | 22.2% | No |
| My undergraduate training prepared me for working with clients from disadvantaged communities | 78 | 41.0% | No |
| Collectively, the skill base of the people I work with means we are well equipped to respond to clients from disadvantaged communities | 79 | 74.7% | Yes, community health agreed more[[13]](#footnote-14) |
| Informal supervision is provided amongst staff on working with people from disadvantaged communities | 81 | 65.4% | No |
| Formal supervision is provided amongst staff on working with people from disadvantaged communities | 81 | 43.2% | No |
| This organisation has policies and procedures that support staff working with disadvantaged communities | 82 | 68.3% | No |
| Staff have access to the tools/resources needed to respond to clients from disadvantaged communities | 82 | 48.8% | No |
| There is a philosophy that guides this organisation’s response to clients from disadvantaged communities | 79 | 59.5% | No |
| There is too much expected of staff in my workplace | 83 | 53.0% | Yes, public health agreed less[[14]](#footnote-15) |
| Most of the time, supervisors provide adequate support when problems arise | 80 | 71.3% | No |
| I am satisfied with my level of job security | 84 | 85.7% | No |
| I am satisfied with my level of pay | 83 | 57.8% | No |
| Staff members are encouraged to undertake training courses | 85 | 77.6% | No |
| Staff members are supported in pursuing qualifications or professional development related to their job | 84 | 77.4% | No |
| This organisation allows staff to take paid leave to undertake training | 82 | 85.4% | No |
| There is a strict emphasis on following policies and procedures in this organisation | 82 | 85.5% | No |
| This organisation allows workers the flexibility they need to meet the needs of clients | 83 | 68.3% | Yes, health promotion agreed less[[15]](#footnote-16) |
| External pressures (e.g. legislation, government regulation, case law, publicity) influence the way this organisation responds to clients from disadvantaged communities | 78 | 84.6% | No |
| The safety of workers is an issue in this service | 83 | 63.9% | No |
| This organisation allows workers enough time to build strong relationships with clients and communities | 81 | 55.6% | Yes, health promotion agreed less[[16]](#footnote-17) |

Table 7: Approximate Proportion of Time Spent on Issues, Groups and Communities

| **Proportion of Time Spent** | **Public Health Issues** | | **Clients from Disadvantaged Backgrounds or Communities** | | **Health Issues With Specific Neighbourhoods or Communities** | |
| --- | --- | --- | --- | --- | --- | --- |
|  | **N** | **%** | **N** | **%** | **N** | **%** |
| 1-20% | 15 | 20.0% | 24 | 34.3% | 32 | 46.4% |
| 21-40% | 12 | 16.0% | 11 | 15.7% | 14 | 20.3% |
| 41-60% | 8 | 10.7% | 16 | 22.9% | 8 | 11.6% |
| 61-80% | 11 | 14.7% | 8 | 11.4% | 5 | 7.2% |
| 81-100% | 29 | 38.7% | 11 | 15.7% | 10 | 14.5% |
| Total | 75 | 100.0% | 70 | 100.0% | 69 | 100.0% |

1. Workforce segments are made up public health, community health and health promotion. [↑](#footnote-ref-2)
2. Up to ten responses were possible per respondent (Total Respondent N = 86) [↑](#footnote-ref-3)
3. Includes social disadvantage and discrimination, income security, social support issues, employment and unemployment issues and accommodation and housing issues (including rental, public and crisis). [↑](#footnote-ref-4)
4. Includes service funding, resources and availability, access to services and waiting lists, service quality and efficacy and knowledge and information about services. [↑](#footnote-ref-5)
5. Includes health of population groups and health issues. [↑](#footnote-ref-6)
6. Includes spatial and environmental issues (including the quality of the environment) and transport issues (for clients, in general or to health services) [↑](#footnote-ref-7)
7. Up to nine responses were possible per respondent (Total Respondent N = 86) [↑](#footnote-ref-8)
8. Up to six responses were possible per respondent (Total Respondent N = 86) [↑](#footnote-ref-9)
9. Up to eleven partnerships were cited by each respondent (Total Respondent N = 86) [↑](#footnote-ref-10)
10. Includes “agree” and “tend to agree” responses [↑](#footnote-ref-11)
11. Workforce segments are made up public health, community health and health promotion. [↑](#footnote-ref-12)
12. χ^2^ (2, N = 82) = 6.026, p = 0.049 [↑](#footnote-ref-13)
13. χ^2^ (2, N = 79) = 12.121, p = 0.002 [↑](#footnote-ref-14)
14. χ^2^ (2, N = 83) = 12.432, p = 0.002 [↑](#footnote-ref-15)
15. χ^2^ (2, N = 82) = 10.638, p = 0.005 [↑](#footnote-ref-16)
16. χ^2^ (2, N = 81) = 7.045, p = 0.030 [↑](#footnote-ref-17)
