# Supplementary material for: Working in disadvantaged communities: What additional competencies do we need?
Source: Aust New Zealand Health Policy. 2009 Apr 24;6:10. doi: 10.1186/1743-8462-6-10 (PMC2684114; doi:10.1186/1743-8462-6-10)
Supplement: Additional file 2 — Competency identification. The report on mapping of competency identification. [file 1743-8462-6-10-S2.doc]

Locational Disadvantage:

Towards a Systematic Education of the Workforce

May 2005

Table of Contents

[Project Rationale 1](#__RefHeading___Toc102934083)

[Population Health Package 3](#__RefHeading___Toc102934084)

[Mapping Exercise 4](#__RefHeading___Toc102934085)

[Recommendations: 16](#__RefHeading___Toc102934086)

[References: 24](#__RefHeading___Toc102934087)

[Appendix 1: 25](#__RefHeading___Toc102934088)

[Appendix 2: 27](#__RefHeading___Toc102934089)

# Project Rationale

In 2001, the Centre for Health Equity Training, Research and Evaluation (CHETRE), University of Western Sydney, University of Sydney and the NSW Department of Health received funding from Public Health Education Research Program (PHERP) Innovation Program to develop a practical approach to assess the capacity of the public health workforce to effectively work with locationally disadvantaged communities.[[1]](#footnote-2) One of the thirteen recommendations made at the end of the project specifically addressed the need to systematically educate and train public health workers in this area. It reads:

**Recommendation #4:**

*“A systematic approach should be developed that enables the current and future public health workforce to receive high quality education in understanding locational disadvantage and evidence-based actions to address this. This should be a logical sequel to understanding the social determinants of health. The approach should include the development of modules within* ***existing continuing education programs offered through professional organisations*** *and* ***in curricula for core professional training*** *for all* ***relevant health professionals****. It is also likely to require the development of* ***“free standing” materials*** *and* ***short course****.”[[2]](#footnote-3)*

After initial discussions, it was decided that mapping the findings of the Locational Disadvantage Project against the Population Health component of the Health Training Package would be a good starting point. Training Packages are sets of nationally endorsed standards and qualifications that “provide the central ‘architecture’” of the VET system.[[3]](#footnote-4) (Please refer to Appendix 1 for more information on VET sector and Training Packages.)

***Why adopt the Training Package approach?***

***The Training Package is viewed as the most potentially influential tool for obtaining systematic education and training of public health workers at different levels.*** First, nationally accredited training courses in the VET sector are mandated to use the Training Packages as a template for their content and assessment. Second, while such courses essentially target VET workers, the very “hands-on” and practical nature of Training Package outcomes provide a means for professionals to translate and complement the more conceptual training they have received in the higher education sector. Third, even for the many training efforts for which national accreditation is not sought, the Training Package remains an authoritative reference source to consult in developing those programs; the Training Package is the result of extensive national consultations with industry and as such provides the best current statement of required industry *standards*. Fourth, while not obligated, it would be ideal for the higher education sector to consider the Training Package and its competency standards as a logical starting point for their curriculum design and development.

***In addition to being a template for different training initiatives, the Training Package is a tool that can potentially assist effective staff management.***  Competencies could be a useful way of defining the work to be done and consequently can become the building blocks or “units of analysis” for understanding, describing, and categorizing labour. They form the best way of easing the translation of business objectives into appropriately applied human capital.[[4]](#footnote-5) Furthermore, competencies could effectively guide many other human resource functions. They could be used as a guide for the development of job descriptions and direct recruitment activities, performance management and training needs assessment.

A summary of the rationale for the use of the Training Package is provided in Figure 1 below.

##### Figure1. Why adopt the Population Health Training Package

Population Health Package

**Influence training at different levels**

Mandated template for nationally accredited training for VET

Practical nature complements conceptual training in higher education sector

Authoritative reference source to guide program and curriculum development for VET and higher education sector

Useful in defining the work and unit of analysis for describing and categorising labour

Translate business objectives into appropriately applied human capital

Guide development of job descriptions, recruitment activities, performance management and training needs assessment

**Assist effective staff management**

Human Capital Alliance (Int’l) Pty. Ltd. was contracted to conduct the mapping exercise. This report briefly summarises the results of this mapping exercise. It begins by providing a description of the Population Health package. It then proceeds to describe the process of mapping and detail the findings. It ends with some recommended courses of action. To foster a better shared understanding, a brief explanation of the different terms used in the report is provided in the box below.

| Clarifying Terminologies ‘Public Health’ and ‘Population Health’ The Locational Disadvantage final report consistently makes reference to the term ‘Public Health’. Yet, the study’s findings are being mapped against the ‘Population Health’ package. There are those that would argue that ‘Public Health’ and ‘Population Health’ do not mean one and the same thing. In the use of these terms in this paper, however, they were both employed to refer to the organised response by society to protect and promote health and to prevent illness, injury and disability and have thus been used interchangeably. Competency, Unit of Competency and Competency standards The term ‘competency’ (also referred to as ‘competence’) is the ability to perform tasks and duties to the standard expected in employment[[5]](#footnote-6). A ‘unit of competency’, on the other hand is an industry specification of performance which sets out the skills, knowledge and attitudes required to effectively fulfil a key function or role in a particular occupation.[[6]](#footnote-7) (Please see Appendix 2 for an example of an actual unit of competency). Collectively, ‘units of competency’ make up the ‘competency standards’. |
| --- |

# Population Health Package

The Population Health package was scoped in 2003, developed in 2004 and submitted to the National Training Quality Council for national endorsement in 2005. Very flexible qualifications have been constructed around a minimum number of compulsory Population Health competencies. This is intended to allow workers from varying backgrounds (eg. support worker with no formal qualifications or health professionals needing to pick up a few Population Health competencies) to tailor their qualifications through judicious unit selection.

These different qualifications are listed below and some specific examples of job outcomes covered by the package are enumerated in Appendix 3.

##### Table 1. Qualifications Covered in the Population Health Package

| Population Health Qualifications |
| --- |
| Certificate II in Population Health |
| Certificate III in Population Health |
| Certificate IV in Population Health |
| Diploma of Population Health |
| Certificate II in Indigenous Environmental Health |
| Certificate III in Indigenous Environmental Health |
| Certificate IV in Indigenous Environmental Health |
| Diploma in Indigenous Environmental Health |

There are a total of 113 competencies being proposed to be in incorporated into the Health Training Package to cover Population Health work. The majority of these would form a bank of elective units of competency that cover generic competencies already covered in other Training Packages and domain-specific units to allow for specialisation into different streams such as health promotion, environmental health, Alcohol and Other Drugs, disability work, social housing, etc. The core competencies for the different qualification levels are outlined in the following table.

##### Table 2. Core competencies for qualifications in the Population Health Package

| **Core competencies** |
| --- |
| Work effectively in the Population Health sector |
| Apply a Population Health Framework |
| Contribute to working with the community to identify health needs |
| Work with the community to identify health needs |
| Contribute to Population Health project planning |
| Plan a Population Health Project |
| Contribute to evaluating a Population Health project |
| Evaluate a Population Health Project |
| Undertake systems advocacy |
| Establish and maintain community, government and business partnerships |
| Build capacity to promote health |
| Establish agents of disease transmission and mode of control (for indigenous environmental health stream only) |
| Provide information and support on environmental health issues (for indigenous environmental health stream only) |

**Note:** It should be noted that in addition to these Population Health core competencies, other units of competency covering more generic competencies in the areas of (1) OHS (*i.e. Follow OHS procedures, Participate in workplace safety practices, Implement and monitor OHS policies and procedures for a workplace, Manage workplace OHS management system*), and (2) understanding/working in organisations (*i.e. Follow the organisation’s policies, procedures and programs, Participate in the work environment, Maintain an effective work environment, Coordinate the work environment*) comprise the compulsory units for the different Population Health qualifications. Given the flexible structure of the Population Health Qualifications Framework, a host of other units of competency to complement these compulsory units may in fact be taken as electives as well.

# Mapping Exercise

## The Process

The mapping exercise was designed first to identify the competencies that would enhance the ability of the workforce to be effective in working in and with disadvantaged communities, and second to assess the extent to which these competencies are covered by the proposed Population Health package.

The competencies were identified by meticulously combing through two of the reports that have been generated by the Locational Disadvantage Project—i.e. *Location, Disadvantage and Health: A review of the literature* and *Locational Disadvantage: Focusing on Place to Improve Health (Final Report).* The literature review was particularly useful in specifying the **knowledge** that is necessary to undertake work using a locational disadvantage approach. The Final Report on the other hand, was able to explore more extensively the factors associated with successful interventions and the required **skills and attitudes** as perceived by the workforce. These were then mapped against the skills, knowledge and attitudes specified by industry as pre-requisites to successful job performance in the form of units of competency of the Population Health package. A complete listing of the required skill, knowledge and attitudes identified from the two reports was then developed and tabled for discussion with some of the project proponents. This was to ensure completeness and accuracy of the information that would be the starting point of the mapping exercise.

The Locational Disadvantage project used an iterative approach in trying to arrive at an understanding of workforce development needs. Apart from surveying available literature, answers to roughly the same questions were obtained through interviews with managers of Area Health Services (AHS), focus group discussions with teams of population health workers and a survey of population health staff across three NSW AHS. The mapping exercise took special note of the competency components (skills, knowledge and attitudes) that were specifically identified in each method of inquiry. With some qualifications, multiple mentions or citing of a competence area across several sources of inquiry were taken to imply greater substance.

## The Findings

### The skills, knowledge and attitudes identified in the Locational Disadvantage Project

Table 3 lists the different skills, knowledge and attitudes identified as required to effectively perform locational disadvantage work. In the table, these were divided into those that relate specifically to locational disadvantage and those that are required in many other domains or areas of work (referred to in the table as ‘generic’). Working collaboratively with others, for example, is a skill that is required in many work areas within and beyond the health industry while predicting, measuring or validating the link between place, disadvantage and health is more specific to a locational disadvantage approach. The table indicates in how many methods of inquiry these were cited.

From a logical standpoint, it seemed that only skills, knowledge and attitudes that have been cited in more than one method of inquiry would be mapped against the Population Health competencies as this ensures some form of validation of their importance to a Locational Disadvantage approach. However, as Table 3 shows, most of the skills, knowledge and attitudes cited in at least two methods of inquiry are more generic competency components. Those that seem to relate more specifically and distinguish a locational disadvantage approach have been identified only at one level (which was in nearly all the cases, identified through the literature review.) It was thus decided that these would be included in the mapping exercise precisely because it is in this area that a significant input could be provided to shape the Population Health package to better suit the needs of the workforce in dealing with locationally disadvantaged communities.

**Table 3. Skills, Knowledge and Attitudes Required in Locational Disadvantage Work**

| **Number of times cited** | **‘Generic’ Knowledge, Skill and Attitude** | **Skills, Knowledge and Attitude Specific to Locational Disadvantage Approach** |
| --- | --- | --- |
| Identified in all four methods of inquiry | - Work collaboratively with others within the same organisation, with other organisations and with the community - Build relationships and partnerships |  |
| Identified in three methods of inquiry | - Conduct needs assessment - Prioritise competing needs of different members of the community and pre-defined objectives of the AHS (e.g. influence the agenda of the AHS, act on local and national policies, elicit commitment to provision of programs and services community found useful) - Inspire confidence and trust - Conduct community consultations - Approach issues from a community perspective and taken on a development approach |  |
| Identified in two methods of inquiry | - Knowledge of characteristics of successful interventions – particularly that they are well-targeted and multi-level or multi-functional - Obtain required funding - Demonstrate political commitment (e.g. gather support from senior managers, find champions within the executive) - Good attitude towards/respects the community |  |
| Identified in one method of inquiry | - Measuring health/illness - Conduct data analysis; Epidemiological skills - Research skills - Design and plan a program/intervention (i.e. understand the target group’s concept of community and the nature and importance of interventions, tailor programs to suit specific needs of the community, extract general characteristics of the population, ensure sufficient time frame, sustainability) - Pick strong committee members - Conduct pilot programs - Evaluate program/intervention - Increase staff and community support and participation (e.g. multiple and ongoing entry and participation points) - Co-ordinate area responses  Capacity building (e.g. training, transfer of skills and knowledge to the community)  - Recognise and use political opportunities - Conduct a debrief - Organisational development - Passion for work - “Thick-skinned” - Sense of humour - Openness - Genuineness - Innovativeness | - Relationship between place of residence and health - Difference between “space” and place - Three levels of forces that operate in the community (macro, meso, micro) and the different types of intervention to address these (upstream, midstream, downstream) - Models of locational disadvantage and health - Characteristics of a locationally disadvantaged area and how these affect health - Mapping location - Monitoring and managing disadvantage - Identifying presence of stressors - Conduct assessment of environment - Explore impact of physical and social contextual factors using methodologies such as ecologic studies, mortality/morbidity rates, contextual and multi-level analysis, comparisons of small well-defined contrasting neighbourhoods - Predicting, measuring or validating the link between place, disadvantage and health – i.e. teasing out the difference between health outcomes associated with a group of people with similar characteristics who live in a particular space (composition) and the impact of the place itself (context) - Know and address the causes for locational disadvantage that lie outside the community |

### Results of Mapping against the Population Health Package

Mapping attempts to match identified skills, knowledge and attitudes against Training Package units of competence. Mapping results fall under four categories of fit or match. These are shown and briefly explained in the illustration below.

On the left side of the quadrant are competency components that map ‘perfectly’ or ‘sufficiently’ to the Training Package. While in both cases ‘contextualising’[[7]](#footnote-8) might be required, development of support materials for competencies that only map sufficiently would be more critical as the identified skills, knowledge and attitude may not map to corresponding competencies in a very straightforward manner.

On the right side of the quadrant are competency components that ‘map poorly’ or ‘do not map at all’ to the Population Health package. It is classified as mapping poorly when the equivalent unit of competency (or a component thereof) has limited scope for adequate coverage of the skill, knowledge and/or attitude it is being matched to. Identified skills knowledge and attitudes that fit in the ‘do not map at all’ category, are obviously not covered even by fragments of units of competency. In these cases, further action would be required – changes might have to be recommended to the Training Package or where the competency components are not able to be addressed within it, action outside the modification of the package may have to be sought. The latter would particularly apply to attitudes, which are more generally not well-handled in the behavioural model that underpins Training Package design and for competencies that are best dealt with in the higher education sector.

**Figure 2. Categories of identified skills/ knowledge / attitude match to the Training Package**

No change to the package or other further action required.

Some changes to the Training Package and/or other further action likely to be required.

| Maps perfectly  The identified competency components match a unit of competency without having to recommend any change. | Maps poorly  There is/are existing relevant unit/s of competency that could be modified to better address the locational disadvantage approach. |
| --- | --- |
| No change to the package required. May be good to develop teaching resources to guide unit customisation.  Maps sufficiently  There is/are broad units of competency that can be *contextualised* to sufficiently cover the requirements for locational disadvantage. | Doesn’t map at all  There are no existing relevant units of competency or the Population Health package is not the place for these skills, knowledge and/or attitudes. |

Results of the mapping exercise for the generic skills, knowledge and attitudes followed by those specifically relating to locational disadvantage are detailed in the following sections.

#### Generic Competency Components

Not surprisingly, most of the generic skills, knowledge and attitudes can be mapped perfectly or at the very least mapped sufficiently to a unit or group of units of competency. In cases where the skills, knowledge and attitude map to more than one competency, it would be a matter of selecting the one most appropriate for the individual workplace requirements (and/or qualification level). Table 4 below enumerates those that map perfectly while Table 5 lists down those that map sufficiently.

Even while these are classified to be a good fit, it would still be useful to develop support materials for individuals who develop and deliver training. It could be a significant challenge for many trainers to work directly with units of competence, especially where they need to discriminate between or combine units of competence. The development of support materials is addressed in the *Recommendation* section of this report.

###### Table 4: Perfect Matches

| **Locational disadvantage skills / knowledge identified as required** | **Equivalent Unit of Competency from the Population Health Package** |
| --- | --- |
| Work collaboratively with others within the same organisation, with other organisations and with the community | *Establish and maintain community, government and business partnerships*  *Participate in networks*  *Maintain effective networks*  *Develop new networks* |
| Build relationships and partnerships | Establish and maintain community, government and business partnerships  *Participate in networks*  *Maintain effective networks*  *Develop new networks* |
| Conduct needs assessment | *Contribute to working with the community to identify health needs*  *Work with the community to identify health needs* |
| Conduct community consultations | Devise and conduct community consultations |
| Approach issues from a community perspective and taken on a development approach | *Apply a community development framework*  *Implement a community development strategy* |

**Table 5. Sufficient Matches**

| **Locational disadvantage skills / knowledge identified as required** | **Equivalent Unit of Competency from the Population Health Package** | **Notes** |
| --- | --- | --- |
| Knowledge of characteristics of successful interventions – particularly that they are well-targeted and multi-level or multi-functional | *Contribute to working with the community to identify health needs* and *Work with the community to identify health needs* have specific elements relating to the conduct of a literature review on relevant intervention programs  *Plan a Population Health project*  **E3/P2.** Strategies most likely to fulfil project goal(s) and objectives are identified on the basis of available literature and suitability, effectiveness, reach and acceptability to stakeholders and target group  **RS.** Appropriate strategies should take into account current trends in health inequalities; current evidence in relating to health and its social determinants, and; socio-economic variables, and measures of health inequality at an area and individual level | Requiring knowledge of evidence-based practice is built-in to the core units for all qualification levels of the package. It is required as essential knowledge for the following competencies  Certs II and III:  *Contribute to working with the community to identify health needs; Contribute to Population Health project planning; Contribute to evaluating a Population Health project*  **Cert IV and Dip:**  *Apply a Population Health framework; Work with the community to identify health needs; Plan a Population Health project; Evaluate a Population Health project* |
| Inspire confidence and trust | Establish and maintain community, government and business partnerships  *Participate in networks*  *Maintain effective networks*  *Develop new networks*  *Utilise specialist communication skills to build strong relationships* | Inspiring confidence and trust is implicitly covered by the different competencies on establishing and maintaining networks, partnerships and relationships. |
| Good attitude towards/respects the community | Contribute to working with the community to identify health needs  *Work with the community to identify health needs*  *Apply a community development framework*  *Implement a community development strategy* | A respectful and responsive attitude towards the community underpins many of the units of competency in the Population Health package. The competencies specified here are some good examples. |
| Prioritise competing needs of different members of the community and pre-defined objectives of the AHS (e.g. influence the agenda of the AHS, act on local and national policies) | *Contribute to policy development*  *Participate in policy development*  *Undertake system advocacy*  *Provide advocacy and representation* |  |
| Obtain and demonstrate political commitment (e.g. elicit commitment to provision of programs and services community found useful, gather support from senior managers, find champions within the executive) | *Undertake system advocacy*  *Provide advocacy and representation* |  |
| Obtain required funding | *Write a grant application*  *Initiate and maintain communication with sponsors/funding organisations* | There are other existing competencies other Training Packages that relate to sourcing required funds. The Population Health Qualifications framework is flexible enough to use these units of competency in place of, or in addition to, those already in the Population Health package. Two such competencies identified in our search are:  (*BSBATSIW515A) Secure funding (from the Business Services Training Package*  *(CUVADM10A) Research and utilise revenue and funding opportunities from the Visual Arts, Craft and Design Training Package)* |

#### Locational Disadvantage-specific components

Mapping the locational disadvantage-*specific* skills, knowledge and attitude have not been as straightforward as mapping the more generic ones. This is due to the fact that specific skills, knowledge and attitudes can be addressed at:

- A *broad* level. At this level, concepts and skills are introduced to the workforce in a very general sense to raise awareness and, in a limited sense, enhance the capacity to take on a locational disadvantage approach.
- A *specialist* level. For those who would like to specialise in locational disadvantage work, it may not be adequate to learn only broad, generic skills, knowledge and attitudes. There may be a need for competencies that specifically address knowledge, skills and attitude at an in-depth level and would differentiate the locational disadvantage worker from other population health workers. In Training Package parlance, these would be taken up as ‘electives’ in a course or qualification.

Table 6 outlines the mapping of the skills, knowledge and attitudes that are more specific to a Locational Disadvantage approach to the Population Health Package at the ‘broader’ and ‘specialist’ worker level. Clearly the correspondence between required skills, knowledge and attitudes and mapped units of competence is more demanding for ‘specialist’ workers.

**Table 6. Mapping of locational disadvantage-specific skills, knowledge and attitudes**

| **Identified knowledge, skills and attitude** | **Related Unit of Competency from the Population Health Package (comments provided where no unit is able to be mapped or only poorly mapped)** | |
| --- | --- | --- |
|  | **Broader worker requirements** | **Specialist worker requirements** |
| Relationship between place of residence and health | *Work effectively in the Population Health sector*  *Apply a Population Health framework* | Does not map at all  This concept could be briefly addressed in some of the core units for Population Health. However, there is no other unit of competency that accommodates this at a more specific level. |
| Difference between “space” and place | *Work effectively in the Population Health sector*  *Apply a Population Health framework* | Does not map at all  This concept could be briefly addressed in some of the core units for Population Health. However, there is no other unit of competency that accommodates this at a more specific level. |
| Three levels of forces that operate in the community (macro, meso, micro) and the different types of intervention to address these (upstream, midstream, downstream) | *Work effectively in the Population Health sector*  *Apply a Population Health framework* | Does not map at all  This concept could be briefly addressed in some of the core units for Population Health. However, there is no other unit of competency that accommodates this at a more specific level. |
| Models of locational disadvantage and health | *Work effectively in the Population Health sector* | Does not map at all  This concept could be briefly addressed in some of the core units for Population Health. However, there is no other unit of competency that accommodates this at a more specific level. |
| Characteristics of a locationally disadvantaged area and how these affect health | *Work effectively in the Population Health sector*  *Apply a Population Health framework*  *Plan a Population Health framework* | Does not map at all  This concept could be briefly addressed some of the core units for Population Health. However, there is no other unit of competency that accommodates this at a more specific level. |
| Identifying presence of stressors | *Work with the community to identify health needs* | Does not map at all  This concept could be briefly addressed in the core units for Population Health. However, there is no other unit of competency that accommodates this at a more specific level. |
| Know and address the causes for locational disadvantage that lie outside the community | *Work effectively in the Population Health sector*  *Apply a Population Health framework*  *Contribute to policy development*  *Participate in policy development*  *Undertake system advocacy*  *Provide advocacy and representation* | Does not map at all  Knowing the causes for locational disadvantage outside the community could be briefly addressed in the core units for Population Health. However, there is no other unit of competency that accommodates this at a more specific level.  Addressing the causes of disadvantage that lie outside the community though could be addressed by advocacy and policy development units. |
| Mapping location | *Work with the community to identify health needs* (with possible slight modification to an element) | Does not map at all  This concept could be briefly addressed in the core units for Population Health. However, there is no other unit of competency that accommodates this at a more specific level. |
| Conduct assessment of environment | *Work with the community to identify health needs* (with possible slight modification to an element)  The assessment of “space”, could be further highlighted. | Maps poorly  This concept could be briefly addressed in the core units for Population Health. However, there is no other unit of competency that accommodates this at a more specific level. |
| Managing disadvantage |  | Does not clearly map too well  There are competencies that relate to design, implementation and evaluation of **population** **health** interventions. However, a component that addresses understanding of interventions relating specifically to managing disadvantage is not readily discernible.  There are other aspects of managing disadvantage which are covered by the package including policy development and advocacy. |
| Monitoring disadvantage | Maps poorly | Does not map at all |
| Explore impact of physical and social contextual factors using methodologies such as ecologic studies, mortality/morbidity rates, contextual and multi-level analysis, comparisons of small well-defined contrasting neighbourhoods | Does not map at all | Does not map at all |
| Predicting, measuring or validating the link between place, disadvantage and health – i.e. teasing out the difference between health outcomes associated with a group of people with similar characteristics who live in a particular space (composition) and the impact of the place itself (context) | Does not map at all | Does not map at all |

As evident from the above table, there are many required skills / knowledge that map sufficiently to the core units of the Population Health Training Package for *the broader population health workforce*. However, for *specialist locational disadvantage workers* most do not map at all.

##### For broader population health workforce

Among those that map poorly for the broader workforce are the skills to ‘map location’ and ‘conduct assessment of environment’. The unit ‘*Work with the community to identify health needs*’ seems a sufficient platform to accommodate these skills. However, the element in the unit ‘collect other relevant data to further define the target group’ is written in a manner that addresses compositional factors but underemphasises contextual factors. There may be a need to propose a slight modification to this element to put equal emphasis on contextual factors, which is essentially what the skills ‘mapping location’ and ‘assessing the environment’ would be about.

‘Monitoring’ and ‘managing’ disadvantage are two other skills that seem to map poorly to the Population Health package. Units of competency relating to identification of community health needs could imply to some extent, the assessment of the level of disadvantage. However, monitoring and managing disadvantage seem to be a more pro-active step—i.e. being aware of the various forms of disadvantage and managing them before they can even impact on health. One may argue that managing disadvantage would essentially involve the design, implementation and evaluation of an intervention and there are such competencies in the Population Health package. However, the existing competencies in the package are restrictive in that they clearly refer to **health** interventions.

‘Explore impact of physical and social contextual factors’ using specific methodologies (e.g. ecologic studies, mortality/morbidity rates, contextual and multi-level analysis, comparisons of small well-defined contrasting neighbourhoods) and ‘predicting, measuring or validating the link between place, disadvantage and health’ do not map at all the current package. They are in a sense inextricably linked to mapping location and assessing environment that was already discussed earlier. However, they seem to likewise involve other higher order skills and knowledge and the Population Health package may not be the ideal platform for these.

##### For specialist locational disadvantage workers

For specialist locational disadvantage workers the mapping that satisfies the competence needs of the broader workforce will not suffice. It would seem that new or modified units of competency will be required. While further consultations are recommended to guide the actual development of new units of competency, it is envisaged that:

- knowledge components (i.e. relationship between place of residence and health; difference between “space” and “place”; three levels of forces that operate in the community and the different types of intervention to address these; the causes for locational disadvantage that lie outside the community models of locational disadvantage and health, and; characteristics of a locationally disadvantaged area and how these affect health) could possibly form a unit of competency that provides an in-depth orientation to locational disadvantage work;
- the skill components ‘identify presence of stressors’, ‘map location’ and ‘conduct assessment of environment’ could possibly form a single unit of competency that relates to identification and assessment of contextual factors; and
- the skill components ‘monitoring’ and ‘managing’ disadvantage may require a suite of new units of competency.

Consultations should perhaps be conducted to investigate more fully the way in which the skills ‘exploring the impact of contextual factors using specific methodologies’ and ‘predicting the link between place, disadvantage and health’ can be better accommodated in the package. Some aspects of it would already be covered by the other proposed units of competency outlined above. But there is perhaps a need to define which aspects of these belong more to the VET sector (and consequently the Population Health package) and which are better addressed in the higher education sector.

# Recommendations:

## On the Population Health Training Package

### Use existing competencies

Nearly all the generic and some of the locational disadvantage-specific competency components (at least at a broad level) consistently mapped back to a limited number of Population Health competencies. The table below lists these competencies and the corresponding knowledge, skills and attitudes they specifically cover.

**Table 7. Population Health x Knowledge, Skills and Attitude Covered**

| **Units of Competency** | **Knowledge, Skills and Attitudes** |
| --- | --- |
| Apply a Population Health Framework / Work effectively in the Population Health Sector | *At a very broad level*: Relationship between place of residence and health, difference between “space” and “place”; three levels of forces that operate in the community and the different types of intervention to address these; Models of local disadvantage and health; Characteristics of a locationally disadvantaged area and how these affect health; Causes for locational disadvantage that lie outside of the community |
| Work with the community to identify health needs / Contribute to working with the community to identify health needs | Conduct needs assessment; Knowledge of characteristics of successful interventions; Good attitude towards/respects the community; Identify presence of stressors (*broad level*); Mapping location (*broad level*); Conduct assessment of environment (*broad level and possibly needs a slight modification for appropriate emphasis*) |
| Plan a Population health project / Contribute to Population Health project planning | Knowledge of characteristics of successful interventions; Characteristics of a locationally disadvantaged area and how these affect health (*broad level*) |
| Undertake systems advocacy / Provide advocacy and representation | Prioritise the competing needs of different members of the community and pre-defined objectives of the AHS; Demonstrate political commitment (e.g. elicit commitment to provision of programs and services community found useful, gather support from senior managers, find champions within the executive); Address the causes for locational disadvantage that lie outside the community |
| Establish and maintain community, government and business partnerships | Work collaboratively with others, with the same organisations, with other organisations and with the community; Build partnerships and relationships; Inspire confidence and trust |
| Participate in networks / Maintain effective networks / Develop new networks | Work collaboratively with others with the same organisations, with other organisations and with the community; Build partnerships and relationships; Inspire confidence and trust |
| Utilise specialist communication skills to build strong relationships | Inspire confidence and trust |
| Apply a community framework / Implement a community development strategy | Approach issues from a community perspective and take on a community development approach; Good attitude towards/respects the community |
| Devise and conduct community consultations | Conduct community consultations |
| Write a grant application | Obtain required funding |
| Contribute to policy development / Participate in policy development | Prioritise the needs of different members of the community and pre-defined objectives of the AHS; Address the causes for locational disadvantage that lie outside the community |

Nearly all of these Population Health units of competency can accommodate the specified locational disadvantage competency components in the above table as they currently stand.

### Modification to existing unit of competency

Among the existing units of competency highlighted in the above table, only the *Work with the community to identify health needs* would possibly require modification to accommodate the requirements of locational disadvantage work. A slight modification to Element #2 of this unit to ensure better emphasis of the need to consider contextual factors when defining the target group would make the unit function well.

### Inclusion of new units of competency

There would still be a need to consider the development of additional units of competency that would cover some of the locational disadvantage-specific requirements in a way that is more suitable for those who wish to specialise in this line of work. As mentioned earlier, these competencies can be roughly grouped as follows:

| **Proposed New Inclusions** | **Knowledge, Skills and Attitudes** |
| --- | --- |
| Orientation to Locational Disadvantage Work | Knowledge components (i.e. relationship between place of residence and health; difference between “space” and “place”; three levels of forces that operate in the community and the different types of intervention to address these; causes for locational disadvantage that lie outside the community models of locational disadvantage and health, and; characteristics of a locationally disadvantaged area and how these affect health) |
| Identify and assess contextual factors that can impact on the health of a community | Identify presence of stressors; Map location and Conduct assessment of environment’ |
| An additional or possibly a suite of new competencies | Monitor and Manage disadvantage |

### Further investigation of other locational disadvantage-specific competency components

The skills ‘exploring the impact of contextual factors using specific methodologies’ and ‘predicting the link between place, disadvantage and health’ have been difficult to map against the Population Health package as at first glance, these seem to belong to the ambit of the higher education sector rather than the VET sector. It may be appropriate to conduct further industry consultations about these skills to clarify further in which way they could be better accommodated in the package

**NEXT STEP:**

We would recommend that some consultation, even within a small group of experts within the industry, be conducted to (1) determine the appropriate modification to element #2 of the unit *Work with the community to identify health needs,* (2) confirm the need for the inclusion of new units of competency and identify their basic components, and (3) further explore how the other locational disadvantage-specific competencies may be better addressed in the Population Health package. Results of this consultation can then form the basis of a request that could be formally communicated to the Community Services & Health Industry Skills Council to consider the necessary modifications to the Training Package.

## Qualifications and Skills Clusters

Based on the knowledge, skills and attitudes that have been identified and mapped against Population Health competencies through this exercise, the qualifications on the following page would be recommended at Certificate levels III and IV. These qualifications should prepare a worker to be able to operate effectively within a Locational Disadvantage community.

| **Certificate III in Population Health (Specialising in Locational Disadvantage Work)**  Total unit requirement: 15   - Work effectively in the Population Health Sector* - Contribute to working with the community to identify health needs* - Contribute to Population Health project planning* - Contribute to evaluating a Population Health project* - Participate in workplace safety issues* - Participate in the work environment* - Participate in networks - Contribute to policy development - Apply a community development framework | **Certificate IV**  **(Specialising in Locational Disadvantage Work):**  Total unit requirement: 18   - Apply a Population Health framework* - Work with the community to identify health needs* - Plan a Population Health Project* - Evaluate a Population Health Project* - Establish and maintain community, government and business partnerships* - Build capacity to promote health* - Undertake systems advocacy* - Implement and monitor OHS policies and procedures for a workplace* - Maintain and effective work environment* - Maintain effective networks - Participate in policy development - Utilise specialist communication skills to build strong relationships - Implement a community development approach - Devise and conduct community consultations - Write a grant application |
| --- | --- |
| 2 Locational disadvantage-specific competencies**   - Orientation to Locational Disadvantage work - Monitor disadvantage | At least 2 Locational disadvantage-specific competencies**   - Assess contextual factors that can impact on community health - Manage disadvantage |
| 4 Electives | 1 Elective |

*Core competencies from Population Health package.

**Would depend on the competencies that are developed as a result of consultation.

Obtaining full qualifications may not always be the most practicable training solution due to limitations such as availability of time or funds. In-service training programs which are based around fewer but absolutely essential basic competencies may be the more feasible alternative. Such training programs for locational disadvantage work (for which no official qualification would be obtained) could arguably be constructed primarily around skills clusters that showcase the more generic competency components which have been more popularly identified through this study. However, while these are invaluable, it would be the locational disadvantage-specific competencies that would differentiate it as a specific domain of work. The skills clusters then should be a combination of the both the more important generic and locational disadvantage-specific competencies and pitched at appropriate levels. Sample skills clusters that can be constructed to guide the development of in-service training programs could look like one of the following:

| **Basic**  Work effectively in the Population Heath sector  Participate in networks  Orientation to Locational Disadvantage work  Monitor disadvantage | **Advanced**  Apply a Population Health framework  Establish and maintain community, government and business partnerships  Assess contextual factors that can impact on community health  Manage disadvantage |
| --- | --- |

| **NEXT STEP:**  The proposed exemplar qualifications have been constructed around competencies that have been identified as necessary for effective performance within a locational disadvantage context. The skills clusters, on the other hand, have been an attempt to draw out the most essential competencies from the proposed exemplar qualifications. Further consultation may be conducted to ensure the appropriateness of these proposed exemplar qualifications, but even more so of the proposed skills clusters, the selection of which was almost arbitrary from a listing of required competencies.  Once exemplar qualifications and skills clusters have been finalised, they may be widely disseminated along with other resource materials to aid development and implementation of different training efforts. This is discussed in greater detail in the next section. |
| --- |

## Development of Resource Materials

Development of resource materials (especially for trainers, but possibly also for learners) to aid the training development, implementation and assessment would be a critical means of influencing the quality and outcome of training efforts. While use of the competency standards themselves (especially for formal assessment) promotes minimum quality standards, resource materials can further enhance the likelihood of achieving uniformity in training outcomes.

Inevitably, there would be a need to develop some materials to supplement the recommended changes to be made to the Population Health Training Package.

**NEXT STEP:**

There may be planned or existing efforts to develop Population Health Training Package resource materials. The outcome of some of these may eventually become officially ‘non-endorsed’ components of the package. As these could potentially guide training activities of providers around the country, there is a need to identify and participate and/or provide input into these endeavours.

Contextualised and expanded versions of the recommended qualifications and/or skills clusters may likewise be developed and disseminated along with other resource materials (such as those that promote the most recent findings and/or publications about locational disadvantage). These would particularly be critical for non-accredited, in-house training for ‘locational disadvantage workers’.

## Values and Attitudes

Among the attitudes identified as necessary for locational disadvantage work, the most critical was a “good attitude or respect for the community”. As mentioned in earlier sections, this underpins many of the competency standards and explicit manifestations are addressed in some competencies (e.g. *Apply a community development framework, Implement a community development strategy*).

Many of the other attitudes identified (e.g. flexibility, passion for work, sense of humour) are difficult to map to any competency standard. While their importance is recognised, there is a question as to whether such attitudes are inherent personal attributes or potentially shaped by training. Even if the latter, the question arises as to how training could effectively shape such personal attributes and whether this is the role of training for locational disadvantage (instead of the responsibility of a broader preparation for employment).

The list of personal attributes that enhance employability identified through this study include commitment, honesty and integrity, sense of humour and adaptability (among others)[[8]](#footnote-9). The Australian Chamber of Commerce and Industry has published a paper entitled “*Employability Skills – An Employer Perspective: Getting What Employers Want out of the Too Hard Basket*”. These ‘employability skills’ are very similar to some of the attitudes identified through the Locational Disadvantage project. How ACCI’s employability skills will be developed and eventually impact on training package development and implementation is still a source of debate in VET circles.

In the meantime, however, there are existing core competencies in the Population Health package (and a range of units of competency on relevant areas such as communication, conflict resolution, time management, working with other cultures, etc.) that would equip an individual with an appreciation and understanding of the context within which he/she has to operate and the competencies required to successfully do so.

## Higher Education

At the moment, there is a strong divide separating the VET and higher education sectors. Ideally, the two should be seen as part of one seamless system. The perceived difference between the two should really be understood as a movement along a continuum with practical and conceptual modes at opposite ends. The same competency components that have been identified by industry that underpin the development of competency standards should then likewise shape curriculum development. Furthermore, the system should facilitate a two-way movement between the two sectors.

The dialogue that needs to take place between the two educational sectors is influenced by many external factors that may not be easily controlled. While many efforts are currently underway to bridge the “two systems”, their seamless integration is undoubtedly still a long way away. In the meantime, small but practical steps within the context of this Locational Disadvantage project may be taken in the hope of influencing educational outcomes in the higher education sector, but also to contribute to this larger process of narrowing the gap between the two.

**NEXT STEP:**

The findings of the Locational Disadvantage Report should be communicated to the higher education sector. A comprehensive listing of the identified skills, knowledge and attitudes required for locational disadvantage work may be particularly useful. This could perhaps be supplemented by the Certificate IV qualification for Population Health that is specialising in Locational Disadvantage which can be used as a framework or touch point from which higher order competencies could be constructed. These would be instrumental in their own mapping processes which could in turn potentially influence curriculum development and assist in envisaging better articulation pathways between the two sectors.

# References:

Locational Disadvantage Consortium, *Locational Disadvantage: focusing on place to improve health,* Sydney: Centre for Health Equity Training Research and Evaluation (CHETRE), UNSW, University of Western Sydney; University of Sydney, New South Wales Department of Health.

Locational Disadvantage Consortium, *Location, Disadvantage and Health: A Review of the Literature,* Sydney: Centre for Health Equity Training Research and Evaluation (CHETRE), UNSW, University of Western Sydney; University of Sydney, New South Wales Department of Health.

Ridoutt, L., et. al (2004). *Calculating demand for an effective public health workforce: A final report for the National Public Health Partnership*.

Ridoutt, L., Santos, T. Dalmulder, M. et. al., *Maximising the impact of competencies on business outcomes* (unpublished).

Australian National Training Authority, http/www.anta.gov.au.

# Appendix 1:

## A Brief Background on VET and Training Packages[[9]](#footnote-10)

### Vocational Education and Training

###### What is it?

Vocational education and training (VET) provides skills and knowledge for work, enhances employability and assists learning throughout life.

In Australia, its foundation was laid in the mid to late nineteenth century, when mechanics’ institutes, schools of mines and technical and working men’s colleges were established to develop the skills of Australia’s working population. For almost 100 years, training was largely for males working full time in traditional trade related industries.

In today’s Australia, VET is offered not only in the public TAFE system, but also through private and community training providers and in secondary schools. It can link to university study options, and provides up to six levels of nationally recognised qualifications in most industries, including high growth, new economy industries.

Training Packages provide the central ‘architecture’ of the VET system. Training Packages are sets of nationally endorsed standards and qualifications for recognising and assessing people’s skills. In industry areas where there are not yet Training Packages, accredited courses are used instead.

###### Who runs it?

Australian, state and territory governments agreed in 1992 to have a national training system, replacing the separate state and territory systems. The Australian National Training Authority and its board were established to advise ministers on national policy and regulation.[[10]](#footnote-11) The ministers meet to make decisions, on the advice of the ANTA Board. State and territory governments implement the decisions ministers make. States and territories are also responsible for registering and monitoring training providers.

Industry is the driving force behind VET, and a network of Industry Skills Councils advises ANTA and its board about current and future industry training needs.

###### Who pays?

Governments provide around half the funds for the national training system – the other half comes from employers and learners themselves.

###### Where do you get it?

Australia has around 4,000 registered training organisations (RTOs) providing nationally recognised training and qualifications. They include TAFE institutes, private training and assessment organisations, enterprises, universities, schools and adult education providers. Training providers have to meet national standards to become RTOs and they are regularly audited for quality.

## What are Training Packages?

Training Packages are sets of nationally endorsed standards and qualifications for recognising and assessing people's skills.

A Training Package describes the skills and knowledge needed to perform effectively in the workplace. They do not prescribe how an individual should be trained. Teachers and trainers develop learning strategies - the "how" - depending on learners' needs, abilities and circumstances.

Training Packages are developed by industry through national Industry Skills Councils or by enterprises to meet the identified training needs of specific industries or industry sectors. To gain national endorsement, developers must provide evidence of extensive consultation and support within the industry area or enterprise.

Training Packages complete a quality assurance process and are then endorsed by the National Training Quality Council (NTQC) on the National Training Information Service (NTIS).

In January 2005, there were 71 endorsed Training Packages. Nine of these were enterprise Training Packages, developed by enterprises for their own unique needs. Training Packages have a set date for review - usually around three years after they are endorsed. Reviews ensure Training Packages remain current to meet industry needs and allow issues that arise during their implementation to be addressed.

# Appendix 2:

## Sample unit of competency

### UNIT HLTPOP301A Work effectively in the Population Health sector

**Unit Descriptor**

This unit assists workers to understand and apply basic principles of Population Health to their work. It also locates the Population Health worker within the larger context of regional and national initiatives and organisations promoting health.

| **ELEMENT** | **PERFORMANCE CRITERIA** |
| --- | --- |
|  |  |
| Apply key principles of Population Health approach to work | - 1. Current and historic concepts of health and its determinants are identified   2. Key principles to a Population Health approach are identified and used to assess current work approach   3. Work approach is modified so as to apply key principles of Population Health |
|  |  |
| Gather information on the organisations within which Population Health work is conducted | - 1. Relevant local, state and national organisations supporting Population Health work are identified   2. Each organisations’ contribution to Population Health work is clarified   3. The role of the worker within the multi-sectoral and multi-strategic approach to Population Health is developed |
|  |  |
| Work within the context of the population health approach | - 1. All population health work reflects consideration of the historical social, political and economic context   2. All worked is checked to ensure it complies with relevant legislative and regulatory frameworks |

|  |  |
| --- | --- |
| **Range Statement** |  |
|  |  |
| *The term population may refer to:* | - General Australian population - Any subgroup within that population identified by age, sex, ethnicity, sexual identity, geographical location, physical environment, lifestyle choices, socio economic status, disability, health status, etc. - Any community defined as collective group of people identified by common values and mutual concern for the development and well-being of their group or geographical area |
|  |  |
| *Context includes:* | - Statutory framework within which work takes place - Historical context of work, e.g. changing attitudes to environmental health, changing approaches to public health - Changing social context of work, e.g. changing government and societal views of environmental health, health promotion and disease prevention aspects of primary health care - Political context, e.g. government policies and initiatives affecting environmental health work - Economic context, e.g. the current economic situation as it relates to and affects environmental health and the subsequent impact on individual and community needs |
|  |  |
| *Concepts/Models of health include:* | - Medical model - Salutogenic model - Social view of health - Academic/professional models of health |
|  |  |
| *Different models of work in the sector may include:* | - Early intervention/ disease prevention - Community development and education - Health promotion - Working with individuals - Working with families and the community - Community funded indigenous environmental health workers - Government health services funded indigenous environmental health workers |
|  |  |
| *Health determinants may include:* | - Political - Biological and genetic factors - Physical environment - Socio-economic factors (e.g. Social gradient, stress, early life, social exclusion, work, unemployment, social support, addiction, food, transport, housing, etc.) - Behavioural - Cultural - Quality of health services |
|  |  |
| *Key principles to a Population Health approach may include:* | - Population focus - Preventive focus with stress on building individual/community’s capacity to control health determinants for a better quality of life - Sensitivity to access and equity issues - Sensitivity to cultural differences - Health as a resource for everyday living and not as end in itself - Health as a result of complex interaction among determinants - Shared responsibility of health - Educational-Ecological approach - Evidence-based approach - Principles of equity and non-discriminatory practice |
|  |  |
| *For some workers, especially those working with remote and / or Indigenous communities, additional principles might include:* | - A holistic and community development approach - Commitment to empowering individuals and the community - Commitment to meeting the needs and upholding the rights of individuals and the community |
|  |  |
| *Different settings for Population Health action may include:* | - Home - School - Hospital - Health services - Community - Work - Transport - Sports and recreation facilities |
|  |  |
| *Examples of local, state and national organisations, and initiatives supporting Population Health work include:* | - Establishments that provide primary health care - Community Clinics - Local public health units - State and national Health departments - Professional and industry associations - Non-government organisations - International health organisations |
|  |  |
| *National charters/declarations include:* | - Health for All by the Year 2000 - Health Promotion: Bridging the Equity Gap |

|  |  |
| --- | --- |
| **Evidence Guide** |  |
|  |  |
| *Critical aspects of assessment:* | - Knowledge of basic principles of Population Health - Knowledge of organisations involved in Population Health in Australia - Ability to discuss a Population Health approach in specific work role context |
|  |  |
| *Essential knowledge:* | - The components of population health including health promotion, environmental health, health protection and prevention of communicable and non communicable diseases - Basic principles of health promotion, e.g. as per Ottawa Charter - Basic knowledge of recent public health strategies and relevant local codes of practice and legislation - National, State and local health initiatives and priorities - Equity issues in population health |
|  |  |
| *Essential skills:* | - Effective communication and interpersonal skills including: - Written or verbal - Gathering information from written sources and/or through verbal questioning - Problem solving - Translating ‘big picture’ information into value at the local level - Contributing effectively to meetings |
|  |  |
| *Resource implications:* | Access to   - A range of Government and non government policy documents and reports and statistics |
|  |  |
| *Method of assessment:* | - Interviewing and questioning - Assignment - Scenarios as a basis for the application knowledge of the population health approach to a specific work role - Scenarios as a basis for the application of knowledge about the foundations of the health of populations to a specific work role |
|  |  |
| *Context of assessment:* | - This unit is most appropriately assessed in the classroom environment - This unit may be undertaken by workers training for a range of jobs in the population health and related sectors. Assessment where practicable should allow for knowledge of population health to be applied to a prospective or actual work role |

# Appendix 3:

## Examples of Job Outcomes Covered by the Population Health Package

| - Indigenous Support Worker - Support Officer - Peer Educators - Indigenous Environmental Health Worker - Healthy Housing Worker - Environmental Health Worker - Indigenous Public Health Officer - Environmental Health Officer - Environmental Health Field Support Officer – Aboriginal Communities - Aboriginal Health Education Officer - Co-ordinator Aboriginal Neighbourhood House - Indigenous Environmental Health Worker - Indigenous Public Health Officer - Coordinator, Aboriginal Neighbourhood House - Environmental Technical Officer - Registry Officer, Cancer Surveillance - Disease Control Officer - Assistant Project Officer - Team Support Worker - Outreach worker, Needle & Syringe Exchange Program - Allied Health Assistant/Community Worker - Data Entry Supervisor - Registry Officer, Cancer Surveillance - Health Sponsorship Coordinator - Outreach Worker | - Gay Education Services Officer - Gay Education & Outreach Officer - Area Health Education Officer - Health Promotion Officer - Health Promotion Project Officer - Schools Program Officer - EdNet Community Educator - Women’s Health Educator - Assistant Community Health Worker, Women’s Health - Team Manager - Assistant Project Officer - Cancer Notifications Coordinator - Immunisation Officer - Immunisation and TB Coordinator - Gay Men’s Education Support Officer - Peer Education Officer - Health Sponsorship Coordinator - Community Development Worker - Community Health Worker - Coordinator, Needle & Syringe Exchange Program - Coordinator, Regional Women’s Health - Health Promotion Coordinator - Area Manager, Health Promotions - Regional Coordinator - Regional Programs Coordinator - Manager, Migrant Health Services - Senior Project Officer - Project Manager - Health Liaison Worker - Assistant Community Health Worker |
| --- | --- |

1. Locational Disadvantage: Focusing on place to improve health (June 2004), p.1. [↑](#footnote-ref-2)
2. Ibid., p. 76 [↑](#footnote-ref-3)
3. http://www.anta.gov.au [↑](#footnote-ref-4)
4. Ridoutt, L. et. al. (2005). Maximising the Impact of Competencies on Business Outcomes. See a working example of this approach in the recently released NPHP document **Calculating demand for an effective public health workforce.** [↑](#footnote-ref-5)
5. http://www.anta.gov.au [↑](#footnote-ref-6)
6. Ibid. [↑](#footnote-ref-7)
7. **Contextualisation** refers to the addition of industry or enterprise specific information to a unit of competency to improve the standards relevance to industry (http://www.anta.gov.au).

   [↑](#footnote-ref-8)
8. ACCI Employability Skills – An Employer Perspective: Getting what employers want out of the too hard basket. [↑](#footnote-ref-9)
9. http://www.anta.gov.au/vetWhat.asp [↑](#footnote-ref-10)
10. ANTA will be dissolved by June 2005. Its functions are to be taken over by the Commonwealth Department of Education. [↑](#footnote-ref-11)
